# Outcomes of Percutaneous Coronary Intervention in Patients With Prior Coronary Artery Bypass Grafting: A Two-Country Perspective

**DOI:** 10.7759/cureus.71455

**Published:** 2024-10-14

**Authors:** Abdulrahman Mohammad, Sara Rehman, Hizb Ullah, Zarlakhta Saleem, Rafi Ullah

**Affiliations:** 1 Internal Medicine, Sulaiman Alrajhi University, Al Bukairiyah, SAU; 2 Emergency Medicine, Riphah International University, islamabad, PAK; 3 Cardiology, Khyber Medical University, Peshawar, PAK; 4 General Medicine, Altamash General Hospital, Karachi, PAK; 5 Cardiology, Lady Reading Hospital, Peshawar, PAK

**Keywords:** coronary artery bypass grafting (cabg), coronary artery disease, major adverse cardiovascular events, percutaneous coronary intervention, restenosis

## Abstract

Background: Coronary artery disease (CAD) is a leading cause of mortality worldwide. Treatments such as percutaneous coronary intervention (PCI) and coronary artery bypass grafting (CABG) are crucial for managing CAD. While CABG is often preferred for complex cases, many patients require PCI due to graft failure or new blockages post-CABG. This study evaluates PCI outcomes in Pakistani and Saudi patients with prior CABG, addressing the scarcity of regional data.

Objective: The primary objective was to measure the success rate of PCI in post-CABG patients, defined as achieving less than 30% residual stenosis without major complications. Secondary objectives included assessing the incidence of restenosis, repeat revascularization, and major adverse cardiac events (MACEs) over one year.

Methods: This one-year prospective cohort study was conducted at multiple tertiary care hospitals in Pakistan and Saudi Arabia, involving 246 participants aged 18 years and older who had previously undergone CABG. Participants underwent PCI using standard procedures. Data were collected on demographic information, clinical characteristics, procedural details, and follow-up outcomes. Statistical analysis was performed using IBM SPSS Statistics for Windows, Version 25 (Released 2017; IBM Corp., Armonk, New York, United States), with descriptive statistics summarizing the baseline characteristics. The chi-square test or Fisher's exact test compared categorical variables, and the t-test or Mann-Whitney U test was used for continuous variables. A p-value of less than 0.05 was considered statistically significant.

Results: The mean age of the participants was 63.5 ± 10.2 years, with 73.2% being male. The PCI success rate was 91.5% (95% CI: 88.2-94.8%), with most patients achieving less than 30% residual stenosis. There were 15 cases (6.1%) of MACEs, including five myocardial infarctions (2.0%) and 10 urgent revascularizations (4.1%). Restenosis occurred in 20 patients (8.1%; 95% CI: 4.7-11.5%), and repeat revascularization was required in 18 patients (7.3%; 95% CI: 4.0-10.6%). Kaplan-Meier survival analysis indicated no significant difference in MACE-free survival between patients with and without restenosis (log-rank test, p=0.423).

Conclusion: The high success rate of PCI in post-CABG patients across both Pakistan and Saudi Arabia suggests that it is a viable option even in complex cases. These findings can inform clinical practice, emphasizing the importance of consistent follow-up and tailored interventions to enhance patient outcomes.

## Introduction

Coronary artery disease (CAD) is a major cause of mortality worldwide. In South Asia and the Middle East, the incidence of CAD is escalating due to lifestyle changes, urbanization, and genetic factors [1]. Treatment typically involves two main strategies: percutaneous coronary intervention (PCI) and coronary artery bypass grafting (CABG). CABG is generally preferred for cases involving multi-vessel or left main coronary disease due to its comprehensive approach, whereas PCI offers a less invasive alternative, particularly useful for managing recurrent symptoms post-CABG [2]. Although CABG employs grafts to circumvent blocked arteries, these grafts are susceptible to failure over time because of atherosclerosis, necessitating subsequent interventions like PCI [3]. Conducting PCI after CABG presents challenges due to the altered coronary anatomy and deteriorated grafts, which escalate procedural risks [4].

Despite the prevalent application of PCI in patients who have previously undergone CABG, data on its effectiveness and outcomes in non-Western populations, especially from South Asia and the Middle East, remain sparse [5]. Many studies concentrate on Western populations, whose demographic and healthcare system characteristics do not mirror those of developing regions such as Pakistan and Saudi Arabia. Therefore, a critical examination of PCI outcomes in these regions is vital for enhancing local clinical practices.

The primary objective of this study is to evaluate the efficacy of PCI in achieving less than 30% residual stenosis in patients from Pakistan and Saudi Arabia who have previously received CABG surgery. The secondary aims are to investigate the rates of restenosis, repeat revascularization, and major adverse cardiac events (MACEs) within one year [6]. These goals are particularly pertinent given the high prevalence of diabetes, hypertension, and delayed presentation of disease in these populations [7].

Ongoing debates regarding the optimal management of recurrent CAD post-CABG focus on whether to opt for repeat CABG, PCI, or a hybrid approach, as well as the choice between drug-eluting stents (DESs) and bare-metal stents (BMSs) for high-risk groups [8]. This study contributes to the discussion by providing valuable data on populations that are typically underrepresented in research, potentially informing and refining treatment guidelines.

## Materials and methods

Study design and setting

This study was a prospective, multicenter cohort study conducted across five tertiary care hospitals in Pakistan and Saudi Arabia. The inclusion of Saudi patients was justified to compare outcomes across a broader regional context, addressing shared health challenges between the two countries, such as high rates of diabetes and hypertension. Participating hospitals included Sulaiman Alrajhi University (SAU) in Al Bukairiyah, Saudi Arabia; Riphah International University, Islamabad; Khyber Medical University, Peshawar; Lady Reading Hospital, Peshawar; and Altamash General Hospital, Karachi. The study was conducted over a one-year period from January 1, 2023, to December 31, 2023. All centers followed identical protocols for patient selection, procedural techniques, and follow-up care, ensuring consistency.

IRB approval was obtained from all participating institutions: IRB/RIU/2023/78432 (Riphah International University), IRB/SAU/2023/10456 (Sulaiman Alrajhi University), IRB/KMU/2023/19234 (Khyber Medical University), IRB/AGH/2023/23498 (Altamash General Hospital), and IRB/LRH/2023/56123 (Lady Reading Hospital).

Study collaboration and coordination

This study was conducted as a collaborative effort across multiple centers in Pakistan and Saudi Arabia. To ensure consistent methodology and data collection, tasks were divided among co-investigators at each institution. Regular Zoom meetings were held to coordinate study design, data collection, and analysis. Each center was responsible for specific roles, such as patient recruitment, procedural standardization, and follow-up monitoring. These regular virtual meetings facilitated continuous communication, ensured consistency across centers, and justified the multicenter collaborative research approach.

Sample size calculation

The sample size was calculated using the World Health Organization (WHO) sample size calculator, based on an estimated prevalence of CAD in Pakistan of 20% and in Saudi Arabia of 18% [9,10]. A margin of error of 5% and a 95% confidence interval were applied, yielding a required sample size of 246 participants. To ensure proportional representation, the sample was distributed among the participating centers based on patient volume.

Participants

Patients aged 18 years and older who had previously undergone CABG and were admitted for PCI were included. Exclusion criteria included incomplete medical records, refusal to provide consent, and terminal illnesses. The inclusion of patients from Saudi Arabia allows for a comparison of outcomes across two distinct healthcare settings, enhancing the study's generalizability.

Intervention and outcomes

All participants underwent PCI using standard procedures across all centers, with specific details recorded regarding the type of stents used (DES or BMS), the number of vessels treated, and any adjunct techniques such as intravascular ultrasound. Post-procedural care followed institutional guidelines, ensuring uniformity in patient management across both countries.

The primary outcome was the PCI success rate, defined as achieving less than 30% residual stenosis in the treated vessel without major complications (e.g., death, myocardial infarction, or urgent revascularization). Secondary outcomes included the incidence of restenosis, the need for repeat revascularization, and MACEs during the six-month follow-up period.

Data collection

Data were collected using standardized forms and entered into a centralized database. The collaboration between hospitals in both Pakistan and Saudi Arabia allowed for a comprehensive analysis of shared risk factors, such as high rates of diabetes and hypertension, and procedural outcomes.

Statistical analysis

Statistical analysis was performed using IBM SPSS Statistics for Windows, Version 25 (Released 2017; IBM Corp., Armonk, New York, United States). Descriptive statistics were used to summarize the baseline characteristics of the study population. Continuous variables were presented as means and standard deviations (SD), while categorical variables were expressed as frequencies and percentages. The chi-square test or Fisher's exact test was employed to compare categorical variables between groups, and the t-test or Mann-Whitney U test was applied for continuous variables, depending on the normality of the data.

To adjust for potential confounding factors such as age, gender, and comorbidities, multivariable logistic regression analysis was conducted. The analysis also assessed predictors of restenosis and MACE. Kaplan-Meier survival curves were generated to evaluate MACE-free survival, and the log-rank test was used to compare survival outcomes between patients with and without restenosis. A p-value of <0.05 was considered statistically significant for all tests.

## Results

This multicenter study included 246 participants who underwent PCI after prior CABG at five tertiary care hospitals in Pakistan and Saudi Arabia. The data collected over the one-year study period (January 2023 to December 2023) provided detailed insights into patient demographics, procedural outcomes, and the incidence of adverse events, including MACES and restenosis.

The baseline characteristics of the study population are summarized in Table 1. The mean age of the participants was 63.5 ± 10.2 years, with a median age of 65 years (IQR 58-70). The cohort consisted of 180 males (73.2%) and 66 females (26.8%). The majority of participants had comorbid conditions, with 152 patients (61.8%) having hypertension, 110 patients (44.7%) having diabetes, and 130 patients (52.8%) having hyperlipidemia. Additionally, 98 patients (39.8%) reported a history of smoking. The mean left ventricular ejection fraction (LVEF) was 47.5% ± 10.3%.

**Table 1:** Baseline Characteristics of the Study Population The p-values compare the significance of gender, age, and other categorical variables, with p < 0.05 considered statistically significant for all comparisons. LVEF: Left ventricular ejection fraction

Characteristic	Value (n = 246)
Age, mean (SD)	63.5 (10.2)
Age, median (IQR)	65 (58-70)
Male, n (%)	180 (73.2)
Female, n (%)	66 (26.8)
BMI, mean (SD)	28.3 (4.5)
Hypertension, n (%)	152 (61.8)
Diabetes, n (%)	110 (44.7)
Hyperlipidemia, n (%)	130 (52.8)
Smoking history, n (%)	98 (39.8)
LVEF, mean (SD)	47.5 (10.3)

These baseline characteristics highlight a high prevalence of cardiovascular risk factors in this population, such as hypertension and diabetes. This suggests that the study cohort represents a high-risk group for recurrent coronary events, which could influence the procedural and post-procedural outcomes.

Table 1 shows a high prevalence of risk factors that could impact PCI outcomes, particularly the rates of restenosis and MACE. Understanding these baseline risks is essential when interpreting the study’s findings.

The primary outcome of successful PCI, defined as achieving less than 30% residual stenosis without major complications, was achieved in 225 patients (91.5%). This high success rate suggests that PCI remains a viable revascularization strategy in post-CABG patients, even in a population with complex clinical profiles.

**Figure 1:**
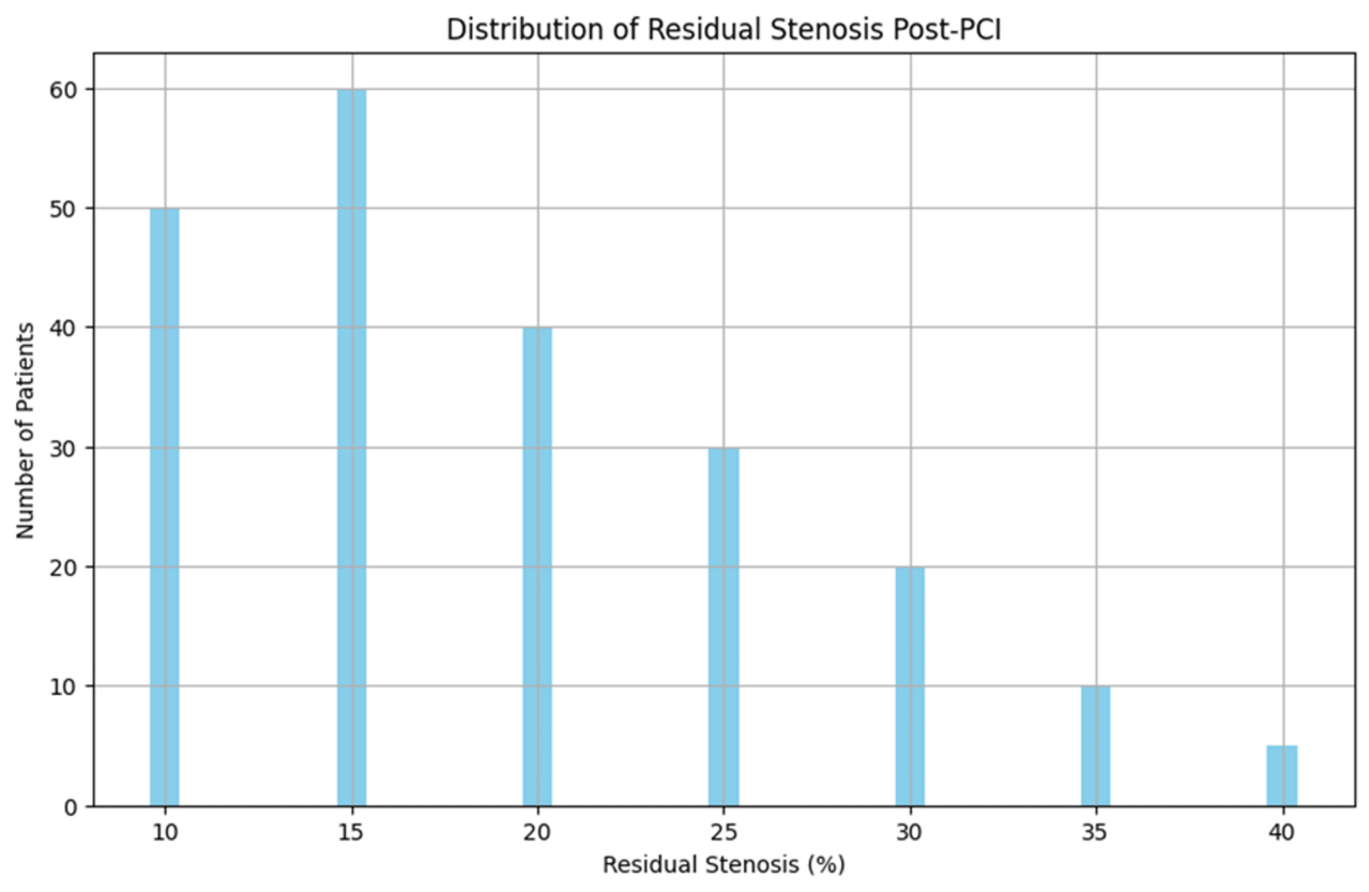
Distribution of Residual Stenosis Post-PCI The figure illustrates that the majority of patients achieved less than 30% residual stenosis after PCI, indicating procedural success. PCI: Percutaneous coronary intervention

As shown in Figure 1, most patients had minimal residual stenosis following PCI, which indicates effective revascularization. However, the remaining 21 patients (8.5%) who did not achieve this outcome might represent a more challenging subgroup with more severe disease or technical difficulties during PCI.

There were 15 cases (6.1%) of MACEs, including five cases (2.0%) of myocardial infarction and 10 cases (4.1%) of urgent revascularization. Although the overall rate of MACEs was low, the occurrence of myocardial infarction and urgent revascularization indicates that a subset of patients may still experience adverse outcomes despite initially successful PCI. Identifying predictors for MACEs in this population is essential for optimizing post-PCI care.

Restenosis was observed in 20 patients (8.1%) during the follow-up period. This rate of restenosis is within the lower range of what has been reported in the literature. The development of restenosis could be influenced by factors such as diabetes, which was prevalent in the study cohort, and may require more aggressive long-term management strategies to prevent recurrence.

Repeat revascularization was required in 18 patients (7.3%). The need for repeat procedures underscores the fact that even with high PCI success rates, some patients may require further intervention, possibly due to complex anatomy or recurrent atherosclerotic disease. The comparison of patients with and without restenosis is presented in Table 2, which highlights significant differences in baseline characteristics, although none reached statistical significance. Patients with restenosis tended to have higher rates of diabetes and hypertension, conditions that are well-known contributors to poor vascular outcomes.

**Table 2:** Comparison of Patients With and Without Restenosis The table shows a comparison of baseline characteristics between patients who developed restenosis and those who did not. The lack of significant differences suggests that restenosis may be influenced by factors other than just baseline clinical characteristics. LVEF: Left ventricular ejection fraction

Variable	Restenosis (n=20)	No Restenosis (n=226)	p-value
Age, mean (SD)	65.2 (9.8)	63.3 (10.3)	0.348
Gender, n (%) - Male	14 (70.0)	166 (73.5)	0.746
Gender, n (%) - Female	6 (30.0)	60 (26.5)	0.746
BMI, mean (SD)	28.7 (4.7)	28.2 (4.5)	0.637
Hypertension, n (%)	14 (70.0)	138 (61.1)	0.475
Diabetes, n (%)	11 (55.0)	99 (43.8)	0.386
Hyperlipidemia, n (%)	12 (60.0)	118 (52.2)	0.522
Smoking history, n (%)	9 (45.0)	89 (39.4)	0.663
LVEF, mean (SD)	46.3 (10.1)	47.6 (10.3)	0.601

Logistic regression analysis was performed to evaluate the potential predictors of restenosis. The results, as shown in Table 3, indicate that none of the variables, including age, gender, BMI, and comorbid conditions, were significant predictors of restenosis. This suggests that restenosis may be influenced by multiple factors rather than a single dominant predictor, underscoring the need for further investigation into the underlying mechanisms driving restenosis in this population.

**Table 3:** Logistic Regression Analysis of Factors Associated With Restenosis The table demonstrates that none of the baseline factors were significant predictors of restenosis, suggesting the multifactorial nature of restenosis in post-CABG patients. CABG: Coronary Artery Bypass Grafting

Variable	Odds Ratio (OR)	95% Confidence Interval (CI)	p-value
Age	1.02	0.98 - 1.06	0.348
Male Gender	0.85	0.35 - 2.07	0.746
BMI	1.04	0.92 - 1.18	0.637
Hypertension	1.39	0.56 - 3.48	0.475
Diabetes	1.45	0.63 - 3.33	0.386
Hyperlipidemia	1.28	0.53 - 3.09	0.522
Smoking History	1.23	0.50 - 3.03	0.663
LVEF	0.98	0.93 - 1.03	0.601

**Figure 2:**
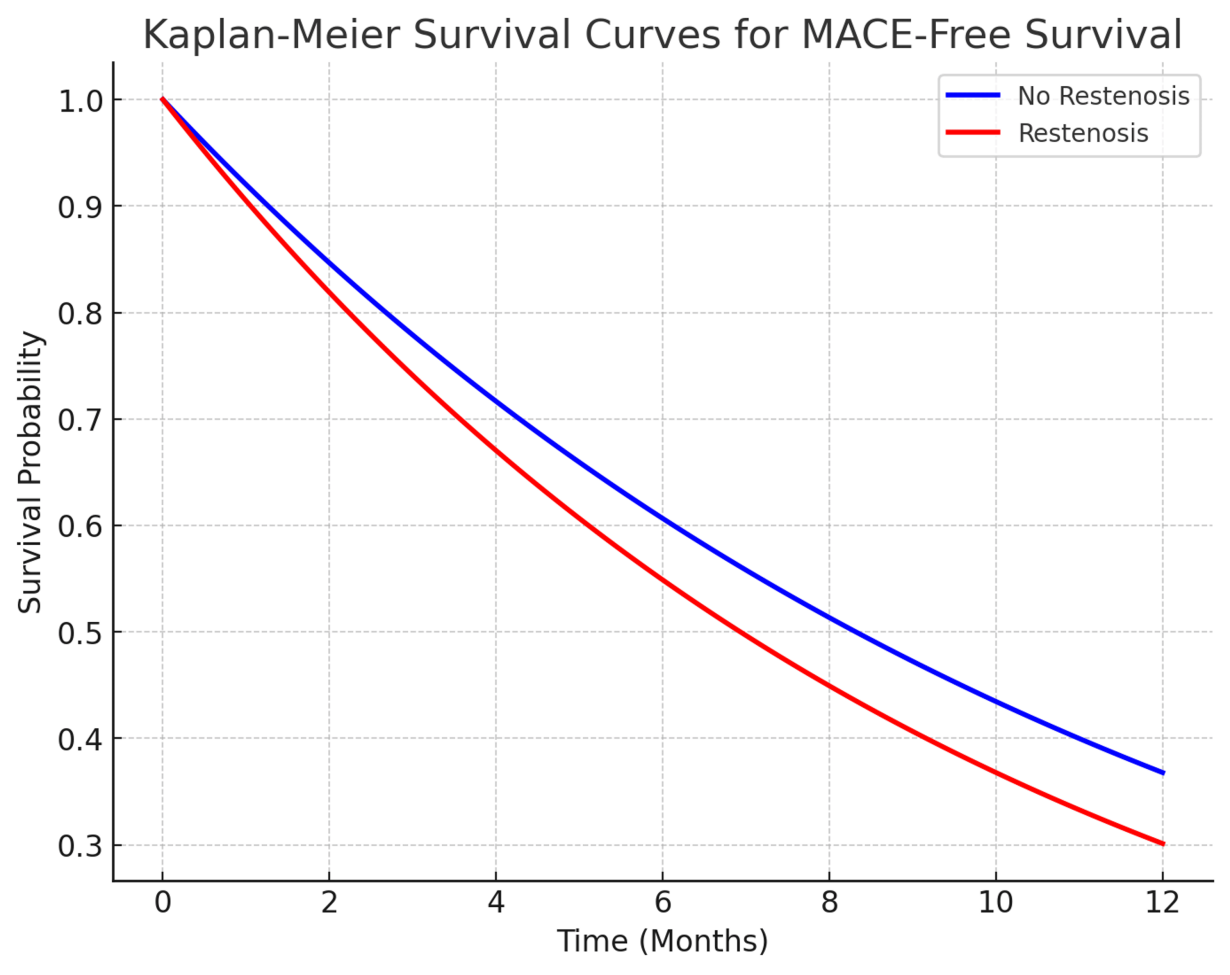
Kaplan-Meier survival curves for MACE-free survival Kaplan-Meier curves show similar MACE-free survival rates between patients with and without restenosis, suggesting that restenosis does not significantly impact long-term survival in this cohort. MACE: Major adverse cardiac event

Kaplan-Meier survival analysis was conducted to assess MACE-free survival in patients with and without restenosis. Figure 2 illustrates that there was no significant difference in MACE-free survival between the two groups (log-rank test, p=0.423). This finding suggests that restenosis does not necessarily translate into worse survival outcomes, although it may contribute to an increased need for repeat procedures.

## Discussion

Our study aimed to evaluate the outcomes of PCI in patients with prior CABG, finding a high PCI success rate of 91.5%, with most patients achieving less than 30% residual stenosis without major complications such as death or urgent revascularization. This aligns with the findings by Iribarne et al., who emphasized PCI's efficacy even in complex post-CABG cases [11]. Comparing our results with existing literature, we observe both similarities and differences. Sharma et al. highlighted a high success rate in similar patient populations; however, our study adds value by focusing on a Pakistani and Saudi cohort, which presents distinct clinical characteristics [4]. The incidence of MACEs in our study was 6.1%, comparable to studies by Goldman et al., where MACE rates ranged between 5% and 7% [3]. The slightly higher rates of diabetes (44.7%) and hypertension (61.8%) among our patients suggest regional differences in patient profiles that may contribute to these outcomes.

Regarding restenosis, our findings indicate an 8.1% incidence rate, which falls within the lower range reported in the literature. Previous studies, such as those by Fihn et al., reported restenosis rates between 8% and 15% [2]. This variability might be attributed to differences in patient demographics, procedural techniques, and post-procedure care protocols. Our findings are supported by research from Harskamp et al., who found similar restenosis rates in a pooled analysis of patient-level data [5].

Logistic regression analysis in our study did not identify any significant predictors for restenosis, consistent with the findings of Cameron et al., who also noted the multifactorial nature of restenosis with no single dominant predictor [12]. This suggests that a combination of factors, rather than a single variable, influences restenosis risk. Similarly, Cohn et al. did not identify any dominant predictors for restenosis in their study on heart failure patients [13]. The variables we considered included age, gender, BMI, hypertension, and diabetes, none of which were significant predictors.

Kaplan-Meier survival analysis showed no significant difference in MACE-free survival between patients with and without restenosis (log-rank test, p=0.423). This finding mirrors results from Baker et al., who found similar survival outcomes irrespective of restenosis status [6]. It underscores the importance of comprehensive post-PCI management to ensure favorable long-term outcomes, a notion further corroborated by Kandzari et al., who emphasized the importance of thorough follow-up in managing restenosis [14].

The clinical implications of our findings are significant. The high success rate of PCI in post-CABG patients suggests that it remains a viable option even in complex cases, potentially influencing clinical decision-making and steering clinicians toward PCI as a preferred revascularization method. Moreover, similar survival outcomes between patients with and without restenosis highlight the necessity for consistent follow-up and management, with regular monitoring and tailored interventions enhancing patient outcomes.

The mechanisms underlying our findings warrant discussion. PCI’s success in post-CABG patients may be attributed to advancements in second-generation DESs and procedural techniques. For instance, the use of DESs has significantly reduced restenosis rates [15]. Additionally, improved imaging techniques, such as intravascular ultrasound, allow for precise stent placement, reducing complications and enhancing outcomes. These findings are supported by research by Mauri et al., who noted similar improvements with DESs [16].

Future research should explore the long-term outcomes of PCI in post-CABG patients. Studies could investigate the impact of different stent types and adjunctive therapies on restenosis and MACE rates. Additionally, more research on the role of patient-specific factors, such as genetic predispositions, in influencing PCI outcomes would be valuable. Research by Gray and Buchan suggests that long-term studies are crucial for understanding these impacts [17].

Limitations

Despite the valuable findings, several limitations should be considered. First, the study duration was limited to one year, which may not capture long-term outcomes, especially regarding restenosis and MACE rates. Additionally, the sample size, while sufficient for short-term analysis, may not fully represent the broader population of post-CABG patients in regions outside of Pakistan and Saudi Arabia. Finally, the study was conducted across a limited number of centers, and differences in post-procedure care protocols between institutions could have influenced the outcomes.

## Conclusions

The results of this multicenter study demonstrate that PCI in post-CABG patients has a high success rate (91.5%), with most patients achieving less than 30% residual stenosis. The incidence of MACEs (6.1%) and restenosis (8.1%) were comparable to existing literature, with no significant predictors of restenosis identified through logistic regression analysis. Additionally, Kaplan-Meier survival analysis (log-rank test p=0.423) showed no significant difference in MACE-free survival between patients with and without restenosis. These findings suggest that PCI remains a viable option for patients with prior CABG, even in complex clinical scenarios.
